# Social determinants of health and emergency department visits among older adults with multimorbidity: insight from 2010 to 2018 National Health Interview Survey

**DOI:** 10.1186/s12889-024-18613-8

**Published:** 2024-04-24

**Authors:** Arum Lim, Chitchanok Benjasirisan, Xiaoyue Liu, Oluwabunmi Ogungbe, Cheryl Dennison Himmelfarb, Patricia Davidson, Binu Koirala

**Affiliations:** 1 Johns Hopkins University School of Nursing, 525 N. Wolfe St, Baltimore, MD USA; 2 University of New York Rory Meyers College of Nursing, 433 1st Ave, NY, USA; 3 University of Wollongong, Northfields Ave, Wollongong, NSW Australia

**Keywords:** Emergency department, Multimorbidity, NHIS, Social determinants of health

## Abstract

**Background:**

Multimorbidity is prevalent among older adults and is associated with adverse health outcomes, including high emergency department (ED) utilization. Social determinants of health (SDoH) are associated with many health outcomes, but the association between SDoH and ED visits among older adults with multimorbidity has received limited attention. This study aimed to examine the association between SDoH and ED visits among older adults with multimorbidity.

**Methods:**

A cross-sectional analysis was conducted among 28,917 adults aged 50 years and older from the 2010 to 2018 National Health Interview Survey. Multimorbidity was defined as the presence of two or more self-reported diseases among 10 common chronic conditions, including diabetes, hypertension, asthma, stroke, cancer, arthritis, chronic obstructive pulmonary disease, and heart, kidney, and liver diseases. The SDoH assessed included race/ethnicity, education level, poverty income ratio, marital status, employment status, insurance status, region of residence, and having a usual place for medical care. Logistic regression models were used to examine the association between SDoH and one or more ED visits.

**Results:**

Participants’ mean (± SD) age was 68.04 (± 10.66) years, and 56.82% were female. After adjusting for age, sex, and the number of chronic conditions in the logistic regression model, high school or less education (adjusted odds ratio [AOR]: 1.10, 95% confidence interval [CI]: 1.02–1.19), poverty income ratio below the federal poverty level (AOR: 1.44, 95% CI: 1.31–1.59), unmarried (AOR: 1.19, 95% CI: 1.11–1.28), unemployed status (AOR: 1.33, 95% CI: 1.23–1.44), and having a usual place for medical care (AOR: 1.46, 95% CI 1.18–1.80) was significantly associated with having one or more ED visits. Non-Hispanic Black individuals had higher odds (AOR: 1.28, 95% CI: 1.19–1.38), while non-Hispanic Asian individuals had lower odds (AOR: 0.71, 95% CI: 0.59–0.86) of one or more ED visits than non-Hispanic White individuals.

**Conclusion:**

SDoH factors are associated with ED visits among older adults with multimorbidity. Systematic multidisciplinary team approaches are needed to address social disparities affecting not only multimorbidity prevalence but also health-seeking behaviors and emergent healthcare access.

## Background

Multimorbidity is defined as the co-existence of two or more chronic conditions [1]. The number of people living with multimorbidity is dramatically increasing worldwide with the growing aging population and improved diagnostic capabilities [2, 3]. According to the pooled data from a meta-analysis of studies published between 2000 and 2021, the prevalence of multimorbidity was 37.2% globally and 43.1% in North America [3]. Since chronic diseases are usually accompanied by aging, 51% of adults aged 60 years and older had multimorbidity in the global population [3]. Multimorbidity is also a problem for middle-aged adults, as multimorbidity stiffly increases after age 50 [4], and 47% of adults 50 years and older have multimorbidity [3]. In support of this, recent studies have extended their focus to individuals aged 50 years and older to investigate multimorbidity and chronic disease burden [5, 6]. Multimorbidity has become a significant health issue because of the increasing complexity of healthcare needs [7]. For example, people with multimorbidity need a multidisciplinary approach to decision-making for the treatment and management of each condition and may have to deal with polypharmacy and communication with multiple health providers [1]. Thus, healthcare providers and researchers have been paying attention to prioritizing the complex needs of care for individuals with multimorbidity [1, 7].

Multimorbidity is associated with adverse health outcomes, such as increased hospital utilization, major health decline, and mortality [8–10]. People with multimorbidity frequently contact general practitioners and visit emergency departments (ED) due to their complex care needs [11, 12]. A study analyzing large electronic health record data in the Netherlands reported that 11% of individuals with multimorbidity had ≥ 12 general practitioner contacts, and 12% had ED visits in a year [11]. However, the group of patients who frequently contact general practice would be distinct from those who visit ED. In the previous study, only 29% of people with frequent general practice contacts had ED visits [11]. This implies that people who do not frequently visit general practice may be more likely to visit ED. In addition, generally, the intensity of ED resource utilization increased with age [13], and people who visited EDs had more chronic conditions and more prescribed medications than the entire multimorbid group [11].

Despite the consistently increased prevalence of multimorbidity for all racial/ethnic groups, the risk for multimorbidity may disproportionally occur due to social disparities. Multimorbidity was related to low educational attainment [14] and was more prevalent among the Black population and less prevalent among Asian and Hispanic populations compared to the White population in the United States (US) [15]. Moreover, emerging evidence has suggested that socioeconomic disadvantages worsen the burden of multimorbidity in older adults [16]. Particularly, low income is consistently associated with not only a higher prevalence of multimorbidity but also worse patient-reported health outcomes in older multimorbid patients [15, 17]. In this context, education, race/ethnicity, and income can be tied up as social determinants of health (SDoH). In general, SDoH consists of five domains: economic stability, education access and quality, healthcare access and quality, neighborhood and built environment, and social and community context [18]. Although gender, education, and health system were the most frequently investigated as SDoH in older multimorbid populations, limited attention is paid to race/ethnicity, socioeconomic status, and political context in the current literature [16]. These indicate that investigating individuals living with multimorbidity and their SDoH factors associated with healthcare access, particularly access to emergency services, is necessary to understand which social context is related to managing multiple chronic conditions leading to ED visits.

Generally, ED visits can be considered as health care needs caused by sudden symptoms, deterioration, or injuries. The frequent complaints leading older people to visit EDs are shortness of breath, chest pain, and lower extremity pain/injury, and in approximately 75% of ED visits, older adults were triaged as urgent/emergent [13]. However, disparities in ED care access and triage processes exist based on race/ethnicity and health insurance status [19]. There is a need to identify and address disparities in emergency healthcare access in older people, which may produce disproportionated health outcomes [19]. Therefore, this study aims to describe the association between SDoH and ED visits among older adults with multimorbidity.

## Methods

### Study design and data source

The study employed a cross-sectional approach to examine the data from 2010 to 2018 National Health Interview Survey (NHIS) conducted by the National Center for Health Statistics (NCHS) [20]. The NHIS is a cross-sectional population-based survey for non-institutionalized civilians aged 18 years or older US adults. The data was gathered by face-to-face interviewing with one randomly selected adult per household for the Sample Adult Module. The interview questions covered healthcare services, behaviors, and health status. Detailed information regarding the design and methodology of NHIS is published elsewhere [20, 21]. This study restricted the study period from 2010 to 2018 to avoid the potential confounding effects of the COVID-19 pandemic that occurred in December 2019 on SDoH and ED visits [22]. This study was exempt from institutional review board review because it used publicly available de-identified data published by the NCHS.

### Sample

#### Inclusion/exclusion criteria

Individuals aged 50 years and older, those who had more than two chronic conditions defined as multimorbidity, and those with available ED visit data were included in this study. The number of chronic conditions was obtained from self-reported disease diagnoses. A total of 10 chronic conditions were selected to define multimorbidity, which were collected throughout the 2010–2018 study period. The selected chronic conditions are parts of conditions defined based on the National Quality Forum Multiple Chronic Conditions framework and aligned with conditions in a study that used this framework to define multimorbidity [23, 24]. Having chronic conditions was identified by the questions asking if the respondents had ever been told by a healthcare professional that they had diabetes, hypertension, asthma, stroke, cancer, arthritis, chronic obstructive pulmonary disease (emphysema or chronic bronchitis), or heart disease (coronary artery disease, myocardial infarction, angina, or other heart conditions), or had been told in the past 12 months that they had weak/failing kidneys or any liver condition. Therefore, participants could have between two and ten chronic conditions.

### Measurements

#### Emergency department visits

The study outcome was one or more ED visits in the previous 12 months. Respondents were asked, “During the past 12 months, how many times have you gone to a hospital emergency room about your own health?” This includes emergency room visits that resulted in hospital admission. The responses were dichotomized as having either one or more instances or none.

#### Social determinants of health

The SDoH variables included in this study were race/ethnicity, marital status, employment and educational status, poverty income ratio, health insurance status, region of residence, and having a usual place to go for medical care when sick. Some variables were defined as dichotomous: marital status (currently married/not married, including never married, divorced, widowed, or separated); employment status (employed/unemployed), insurance status (insured/uninsured), and have a usual place to go for medical care when sick–a proxy for healthcare access (yes/no). Race/ethnicity was categorized as (non-Hispanic White, non-Hispanic Black, non-Hispanic Asian, and Hispanic). Educational status was categorized by ≤ high school, some college, and ≥ bachelor’s degree. The poverty income ratio (PIR) was used as a proxy of financial status. The midpoint of an individual’s family income was divided by the poverty threshold for the year. The variable was then categorized as < 1, between 1 and 1.99, and ≥ 2. A PIR less than one means that the individual income is below the federal poverty level, a PIR between 1 and 1.99 indicates the income is between 100% and 199% of the poverty level, and a PIR greater than two means that the income is more than 200% of the poverty level. The region of residence had four categories: northeast, midwest, south, and west. Perceived health status was categorized on a five-scale from “excellent” to “poor.”

#### Covariates

We included three covariates: age in years (measured as a continuous variable, further categorized as 50–64 years or 65 and older years), sex (categorized as male or female), and the number of chronic conditions. Based on the rationale that having two or more chronic conditions defines multimorbidity, and having three or more is considered complex multimorbidity [4], the number of chronic conditions was categorized into 2, 3–4, or ≥ 5 conditions. The category will help stratify the participants by the severity of multimorbid conditions [25, 26].

### Statistical analysis

This study merged NHIS data from 2010 to 2018 and applied sampling weights according to NCHS guidelines [27]. Sociodemographic characteristics were presented using descriptive statistics, mean and standard deviation, and percentage. Differences and associations in characteristics between respondents with more than 1 ED visit and those without ED visits were examined by survey-weighted t-tests for continuous variables and chi-square tests for categorical variables.

This study used survey-weighted multivariable logistic regression to test the association between SDoH and ED visits within the previous 12 months in people with multimorbidity. Model 1 included multiple SDoH factors, including race/ethnicity, education, income, employment, insurance, marital status, and region of residence, to show the association of each SDoH factor with ED visits, adjusting for all other SDoH effects. Age and sex variables were added in Model 2, and the number of chronic conditions in Model 3. Cases with at least one missing data in any variable were deleted from the analysis, which may cause bias if the missing is not random [28]. Statistical significance was set as a two-sided α < 0.05. All statistical analyses were conducted using the Stata© SE statistical software.

## Results

### Sample characteristics

A total of 28,917 respondents living with two or more of the 10 chronic conditions were included in the analysis. Among them, 68% (*n* = 19,661) had no ED visit, while the remaining 32% (*n* = 9,256) had at least one ED visit in the previous 12 months. The participants’ mean age (± SD) was 68 (± 10.7). The ED visits group had more female participants (58.5%) and adults who were not married (64.7%) than the no ED visit group (56.0% and 56.0%, respectively). The ED visits group had more non-Hispanic Black people (15.8% vs. 11.4%) and Hispanic participants (8.2% vs. 7.2%) than the no ED visit group and were more likely to have a high school or lower education (53.6% vs. 46.8%) and be unemployed (77.1% vs. 67.9%). Moreover, they were more likely to have low PIR (22.3% vs. 13.5%) and more likely to have a usual place for medical care (97.8% vs. 97.0%) than their no ED visit counterparts. Perceived health status was poorer (19.2% vs. 7.5%) in the ED visit group. Health insurance status did not differ between the two groups. In terms of chronic conditions, the most frequently reported condition was hypertension (83.0%), but there was no significant difference between the two groups. In both groups, heart disease (52.0% vs. 39.1%, *p* <.001) and diabetes (38.9% vs. 35.8%, *p* <.001) followed, and there were significant differences in the prevalence of the chronic conditions between the ED visit group and no ED visit group. COPD was the fourth most prevalent chronic condition in the ED visit group, with a higher prevalence than in the no ED visit group (37.4% vs. 24.9%, *p* <.001). However, cancer was the fourth most prevalent chronic condition in the no ED visit group, which was the only disease with a significantly higher prevalence in the no ED visit group than the ED visit group (32.1% vs. 30.4%, *p* <.001). The average numbers of chronic conditions (± SD) were 3.3 ± 1.31 in the ED visit group and 2.7 ± 0.99 in the no ED visit group, and the proportions of having more than five conditions were 9.7% and 6.3%, respectively. The characteristics of the study population can be found in Table 1.

**Table 1:** Sociodemographic characteristics of older adults with multimorbidity by the number of ED visits (*n* = 28,917)

Characteristics	Total	No ED visits	≥ 1 ED visits	*P*
**Weighted, n**	9 589 647	6 583 984	3 005 662	
**Unweighted, n**	28 917	19 661	9 256	
**Age (years, mean ± SD)**	68.04 ± 10.66	68.09 ± 9.88	67.94 ± 10.80	0.339
50–64	40.03	39.01	42.25	< 0.001
≥65	59.97	60.99	57.75	
**Sex (%)**				0.001
Female	56.82	56.04	58.53	
Male	43.18	43.96	41.47	
**Marital status (%)**				< 0.001
Not married	58.70	55.96	64.69	
**Race/ethnicity (%)**				<0.001
Non-Hispanic White	76.44	77.90	73.25	
Hispanic	7.50	7.16	8.22	
Non-Hispanic Black	12.79	11.42	15.81	
Non-Hispanic Asian	2.41	2.73	1.69	
Other races	0.87	0.79	1.03	
**Education (%)**				< 0.001
≥ Bachelor’s degree	22.18	24.00	18.19	
Some college	28.89	29.18	28.25	
≤ High school	48.93	46.82	53.55	
**Poverty-income ratio (PIR)** ^*^ **(%)**				< 0.001
PIR ≥ 2.00	59.08	63.20	50.05	
PIR 1-1.99	24.71	23.34	27.70	
PIR < 1	16.21	13.46	22.25	
**Employment status (%)**				< 0.001
Unemployed	70.79	67.93	77.08	
**Health insurance status (%)**				0.934
Uninsured	4.31	4.30	4.33	
**Have usual place for medical care (%)**	97.22	96.95	97.82	< 0.001
**Region of residence (%)**				
Northeast	17.20	17.1	17.44	0.007
Midwest	24.36	23.85	25.50	
South	39.39	39.43	39.30	
West	19.04	19.63	17.76	
**Perceived health status (%)**				< 0.001
Excellent	6.81	8.27	3.61	
Very Good	21.39	24.77	14.00	
Good	35.27	37.25	30.94	
Fair	25.35	22.20	32.24	
Poor	11.18	7.51	19.21	
**Chronic conditions (%)**				
Diabetes	36.76	35.79	38.89	< 0.001
Hypertension	82.96	83.05	82.75	0.568
Asthma	25.36	23.95	28.42	< 0.001
Stroke	12.55	10.23	17.63	< 0.001
Cancer	31.54	32.05	30.44	0.022
Arthritis	16.88	14.47	22.15	< 0.001
COPD^†^	28.8	24.86	37.44	< 0.001
Heart disease^‡^	43.13	39.10	51.95	< 0.001
Weak/failing kidneys in 12 m	8.22	6.21	12.64	< 0.001
Any liver conditions	3.86	3.24	5.19	< 0.001
**Number of chronic conditions** **(mean ± SD)**	2.90 ± 1.18	2.73 ± 0.99	3.27 ± 1.31	< 0.001
2	48.27	54.30	48.27	<0.001
3–4	42.09	39.37	42.09	
≥ 5	9.65	6.33	9.65	

SD, standard deviation; ED, emergency department; COPD, chronic obstructive pulmonary disease Weighted sample demographic and characteristics were presented and used for inferential statistics. ^*^PIR < 1 = below poverty level; PIR 1-1.99 = between 100-199% above poverty level; PIR ≥ 2 = 200% or above poverty level. ^†^COPD is defined as ever being told they had COPD, emphysema, or chronic bronchitis. ^‡^Heart disease is defined as ever being told they had coronary heart disease, heart attack, or other heart condition/disease

### Social determinants of health on ED visits

The variables representing SDoH (marital status, race/ethnicity, education, financial status, region of residence, and usual healthcare access) were included in Model 1 without adjusting for other covariates. After adjusting for age and sex in Model 2, the associations of SDoH with ED visits were still preserved. Model 3 adjusted for the number of chronic conditions, which was a proxy of the severity of diseases, and all associations were still significant as Model 1 and 2. People who were not married (Adjusted Odd Ratio [AOR]: 1.19, 95% Confidence Interval [CI]: 1.11–1.28), non-Hispanic Black people (AOR: 1.28, 95% CI: 1.19–1.38), had high school education or less (AOR: 1.10, 95% CI: 1.02–1.19), had lower PIR (AOR: 1.44, 95% CI: 1.31–1.59), were unemployed (AOR: 1.33, 95% CI: 1.23–1.44), and had a usual place for medical care (AOR: 1.46, 95% CI: 1.18–1.80) were more likely to visit ED at least once in the prior 12 months, compared to their reference groups. The adjusted findings are presented in Table 2.

**Table 2:** Logistic regression analyses of the associations between social determinants of health and having ≥ 1 ED visit in the prior 12 months among older adults with multimorbidity (*N* = 28,917)

Social determinants of health	Model 1^a^	Model 2^b^	Model 3^c^	
	AOR (95% CI)	AOR (95% CI)	AOR (95% CI)	
**Age (years)**				
50–64	-	Ref	Ref	
≥ 65	-	0.76 (0.71–0.82) *	0.77 (0.71–0.82) *	
**Sex**				
Female	-	Ref	Ref	
Male	-	1.01 (0.95–1.07)	1.01 (0.95–1.08)	
**Marital status**				
Currently married	Ref	Ref	Ref	
Not married	1.20 (1.12–1.28) *	1.22 (1.14–1.31) *	1.19 (1.11–1.28) *	
**Race/ethnicity**				
Non-Hispanic White	Ref	Ref	Ref	
Hispanic	1.07 (0.97–1.18)	1.06 (0.96–1.17)	1.10 (1.00–1.22)	
Non-Hispanic Black	1.28 (1.19–1.38) *	1.25 (1.16–1.35) *	1.28 (1.19–1.38) *	
Non-Hispanic Asian	0.66 (0.55–0.79) *	0.67 (0.56–0.80) *	0.71 (0.59–0.86) *	
Non-Hispanic Other	1.28 (0.93–1.76)	1.23 (0.89–1.69)	1.08 (0.78–1.50)	
**Education**				
≥ Bachelor’s degree	Ref	Ref	Ref	
Some college	1.11 (1.02–1.20) *	1.09 (1.00–1.18) *	1.05 (0.97–1.14)	
≤ High school	1.14 (1.06–1.23) *	1.15 (1.06–1.24) *	1.10 (1.02–1.19) *	
**Poverty income ratio (PIR)** ^†^				
PIR ≥ 2.00	Ref	Ref	Ref	
PIR 1-1.99	1.28 (1.18–1.39) *	1.26 (1.16–1.37) *	1.19 (1.10–1.30) *	
PIR < 1	1.70 (1.55–1.86) *	1.59 (1.44–1.75) *	1.44 (1.31–1.59) *	
**Employment status**				
Employed	Ref	Ref	Ref	
Unemployed	1.35 (1.26–1.45) *	1.52 (1.41–1.64) *	1.33 (1.23–1.44) *	
**Health insurance status**				
Insured	Ref	Ref	Ref	
Not insured	1.02 (0.87–1.18)	0.92 (0.79–1.07)	1.00 (0.85–1.16)	
**Have a usual place for medical care**				
No	Ref	Ref	Ref	
Yes	1.54 (1.26–1.89) *	1.57 (1.28–1.93) *	1.46 (1.18–1.80) *	
**Region**				
Northeast	Ref	Ref	Ref	
Midwest	1.08 (0.98–1.19)	1.08 (0.97–1.19)	1.06 (0.96–1.17)	
South	0.95 (0.87–1.03)	0.94 (0.86–1.03)	0.91 (0.83–1.00)	
West	0.95 (0.85–1.05)	0.95 (0.85–1.05)	0.94 (0.84–1.05)	
**Number of chronic conditions**				
2	-	-	Ref	
3–4	-	-	1.76 (1.65–1.88) *	
≥ 5	-	-	3.56 (3.23–3.92) *	

a. Model 1: Adjusted for other social determinants of health b. Model 2: Adjusted for other social determinants of health, age, and sex. c. Model 3: Adjusted for other social determinants of health, age, sex, and number of chronic conditions *Denotes statistical significance (*P* <.05) ED, emergency department; AOR, adjusted odds ratio; CI, confidence interval; PIR < 1 = below poverty level; ^†^PIR 1-1.99 = between 100-199% above poverty level; PIR ≥ 2 = 200% or above poverty level. Results are weighted

## Discussion

This study presented multiple SDoH factors associated with ED visits among older people with multimorbidity. Particularly, people who were non-Hispanic Black people, not married, had poor financial conditions, and lower education levels showed higher odds of ED visits.

This study demonstrated racial/ethnic disparity in ED visits among older adults with multimorbidity. In this study, non-Hispanic Black people were more likely to have at least one ED visit than other racial/ethnic populations, while non-Hispanic Asian people were less likely to do so. Since multimorbidity was more prevalent among Black people and less prevalent among Asian people [15], this study result implied that race/ethnicity potentially deepened existing multimorbidity disparities through emergent healthcare access disparities. Similarly, the correlation between race/ethnicity and ED visits may indicate existing disparities in multimorbidity status. A study pointed out that Black individuals had a similar prevalence of multimorbidity as other groups who were 5–10 years older, and there was no significant change in multimorbidity prevalence between the Black and White populations from 1999 to 2018 [15]. Factors contributing to this may include the accumulated effect of the health experiences with chronic conditions in early life, producing a gap in older age and leading to higher odds of ED visits. However, since our study results were produced after other SDoH were adjusted, such as education levels and financial status, it needs to be investigated in further research to explore the other possible reasons for racial/ethnic disparity in ED visits. After exploring the mechanisms of deepening health disparities in the treatment continuum, it is necessary to eliminate the disparities led by early-onset chronic conditions and care processes through healthcare intervention and policy.

The findings reported that people who were not married showed higher odds of ED visits than those who were married. Since this study merged responses indicating not married, such as divorced, separated, and bereaved, as unmarried participants, people categorized as not married may include those living alone. Thus, they might lack caregivers, which increases their self-care burden, as well as available resources, such as health insurance, given that married people are more likely to have private insurance than unmarried people [29]. Moreover, married people are more likely to have social support than those who are not given that marital status is often used as a proxy for informal social support [30, 31]. This finding aligns with previous studies that older adults living alone had higher odds of ED admission [32, 33], and people with multimorbidity who live alone had significantly longer inpatient days after ED admissions than those without multimorbidity [32]. These findings may be supported by the fact that multimorbid people have more care needs due to the complexity of care, as well as greater disease and symptom burdens [34, 35]. This study also showed that the lowest education level was associated with higher odds of ED visits than the highest. This can be related to the gap between high healthcare needs and capacity for self-management, given that education is associated with the activation of self-management in patients with multimorbidity [36, 37]. Moreover, lower education level was also related to the greater impact of multimorbidity on activities of daily living and mental health, which may affect self-care [35]. Therefore, since people with multimorbidity have higher needs for self-care, SDoH factors related to self-care, such as marital status and education levels, may explain the higher odds of ED visits in unmarried and lowest education-level participants. Based on this finding, improving self-care and health literacy and implementing social support models in older multimorbid populations may prevent worsening health conditions, reducing ED visits.

The study demonstrated that people who are unemployed and have lower PIR levels have higher odds of ED visits. However, these findings need to be cautiously interpreted due to the cross-sectional study design. It could be explained that older people living with multimorbidity who visit EDs at least once in the previous 12 months are more likely to lose or quit their jobs or have not gotten a chance to be hired due to their poor health conditions. It also influences poverty levels, making lower PIR associated with ED visits. Moreover, this study used the variable ‘having a usual place for medical care’ as a proxy of health care access, one of the SDoH factors. Since it is assumed that people more likely to visit EDs would have worse health conditions, they need to receive regular follow-ups to assess and manage their health conditions. It is reported that multimorbid people are likely to spend more on healthcare costs, consequently making them more vulnerable to cost-related non-adherence to recommended treatment, resulting from financial strain [38, 39]. A study found that more than one-third of participants living with multimorbidity had not sought medical care or purchased medication due to cost [40]. Non-adherence to recommended general healthcare visits and medication may lead to worsened symptoms in multimorbidity populations, which may result in higher odds of ED visits. In this context, their financial burden should be assessed and managed to prevent non-adherence to treatments and management of their multiple chronic conditions, leading to unplanned ED visits due to sudden deterioration. What is apparent is, however, that EDs are commonly the safety net of society [41]. A study reported that the most represented reasons for referral to social work in ED were financial concerns and resource counseling [41]. This indicates that EDs may play a role as the safety net to prevent deepening the disparities in SDoH among people with multimorbidity.

Lastly, the COVID-19 pandemic has tremendously influenced not only people’s SDoH, including employment status and socioeconomic level [42], but also ED visits, such as the number of ED visits and hospital admission rate from ED [43]. Although this study does not explain the impact of the COVID-19 pandemic on the association between SDoH and ED visits, we propose future studies that examine changes in the context of SDoH and emergency healthcare access pre- and post-pandemic.

### Limitations

This study has significance, given that it used large-scale, nationally representative data to strengthen generalizability. Moreover, to our knowledge, this is the first study to examine the association between SDoH and ED visits in older multimorbid populations. However, this study acknowledges the following limitations. First, multimorbidity criteria did not include mental health problems, including substance use. If this study included mental health problems as multimorbidity criteria, the prevalence of multimorbidity would increase, which may influence the results. Moreover, since people with mental health problems are more likely to have multimorbidity [44, 45], mental health may be associated with both SDoH and ED visits, producing a confounding effect. Thus, it may be beneficial to include mental health problems in regression models or multimorbid criteria for future studies to test whether they influence the association between SDoH and ED visits. In addition to mental health problems, some other possible chronic diseases that are common in middle and older age groups should also be comprehensively considered in further studies. Second, the study outcome (ED visits) and inclusion criteria (multimorbidity) were self-reported, which may yield recall bias and information bias. A more systematic way to collect clinical data, such as data extraction of health care utilization and disease diagnosis codes from electronic health records, may reduce the risk of bias in further studies. Third, the cross-sectional approach in this study could not test a causal relationship between SDoH and ED visits. Thus, longitudinal studies are needed to examine whether SDoH affects ED visits to rule out reverse causality. In addition, this study did not cover all domains of the SDoH definition (e.g., neighborhood/built environment and social/community) and adjusted for other SDoH to examine each SDoH effect on ED visits. Addressing all SDoH domains inclusively and considering the intersectionality of SDoH would be beneficial in examining the additive effects of SDoH on ED visits.

Lastly, the number of chronic conditions was adjusted in the final regression model to account for the potential confounding effect of the severity of overall chronic conditions on the association between SDoH and ED visits. Usually, the Charlson Comorbidity Index (CCI) or the number of diseases is used as a proxy to adjust for the severity of overall chronic conditions [46, 47]. However, both CCI and the number of comorbidities may not be perfectly fitted with this study as a covariate since CCI was developed as a predictor of 1-year mortality and burden of disease [48], and the number of diseases cannot account for how comorbidities interact [46], although it is assumed that increasing the number of diseases may lead to increased overall severity. Unfortunately, the NHIS dataset in this study did not cover all the diagnoses to calculate CCI, so this study included the number of diseases in Model 3 as a proxy of the severity of the overall condition. However, it should be interpreted cautiously regarding the confounding effect of the severity of conditions in case it does not reflect the severity of the health condition very well. A few studies have tried to develop proper tools to measure the severity of multimorbid conditions, such as the multimorbidity interaction severity index [46]. However, this preliminary tool still needs to be verified for its reliability and validity in multiple populations [46]. Thus, a proper measure for the severity of multimorbid conditions is necessary to be developed to examine the association between SDoH and ED visits in older multimorbid populations more precisely.

### Implications

Multimorbidity is increasing, and individuals with multimorbidity are high utilizers of health care. Prevention and management of multimorbidity is now a key priority globally. There is increasing attention toward studies focusing on etiology, epidemiology, and risk factors [1], yet there is still limited evidence to support effective healthcare interventions [4]. As there is a need for increased awareness of multimorbidity, innovation, and optimization of the use of existing resources, understanding existing disparities of emergent care needs and vulnerable groups can help determine which factors or combinations of factors are most important to target. The findings of this study underscore the importance of not only addressing early-life disparities contributing to developing multimorbidity but also the SDoH that influences health status and emergent care needs. Particularly, increased health screening and assessment in primary care settings is needed for racial/ethnic minority populations who have the disadvantage of emergent care access. Moreover, unemployed status and worsened financial burden, which may hinder treatment adherence, should be addressed in the context of the treatment continuum among multimorbid people to prevent unplanned worsening symptoms and hospitalization. Lastly, the self-care burden and need for social support in older multimorbid groups need to be paid more attention to mitigate the SDoH effect on emergent healthcare access.

## Conclusions

In conclusion, this study demonstrated that SDoH are associated with increased ED visits among older adults living with multimorbidity. Systematic multidisciplinary team approaches are needed to address social disparities affecting multimorbidity prevalence, health-seeking behaviors, and emergent healthcare access. Therefore, researchers, healthcare practitioners, and policymakers should pay attention to addressing the social disparities by improving the management of chronic health conditions and promoting health equity.

## Data Availability

The datasets generated and/or analyzed during the current study are available in the NHIS repository: https://www.cdc.gov/nchs/nhis/data-questionnaires-documentation.htm.

## Abbreviations

AOR: Adjusted Odds Ratio
CCI: Charlson Comorbidity Index
CI: Confidence Interval
ED: Emergency Department
NCHS: National Center for Health Statistics
NHIS: National Health Interview Survey
PIR: Poverty Income Ratio
SD: Standard Deviation
SDoH: Social Determinants of Health
US: United States
